# Characteristics of Adults’ Use of Facebook and the Potential Impact on Health Behavior: Secondary Data Analysis

**DOI:** 10.2196/ijmr.9554

**Published:** 2018-06-14

**Authors:** Kelly Bosak, Shin Hye Park

**Affiliations:** ^1^ School of Nursing University of Kansas Medical Center Kansas City, KS United States

**Keywords:** social media, health promotion, health behavior, adults

## Abstract

**Background:**

Social influences are a primary factor in the adoption of health behaviors. Social media platforms, such as Facebook, disseminate information, raise awareness, and provide motivation and support for positive health behaviors. Facebook has evolved rapidly and is now a part of many individuals' daily routine. The high degree of individual engagement and low attrition rate of this platform necessitate consideration for a potentially positive impact on health behavior.

**Objective:**

The aim of this paper is to investigate the use of Facebook by adults. Knowledge is limited to the unique characteristics of Facebook users, including time spent on Facebook by adults of various age groups. Characteristics of Facebook users are important to understand to direct efforts to engage adults in future health behavior interventions.

**Methods:**

Institutional Review Board approval was obtained for this secondary analysis of existing de-identified survey data collected for the Pew Research Center. The sample included adults age 18-65 years and above. Binomial logistic regression was performed for the model of age group and Facebook use, controlling for other demographics. A multinomial logistic regression model was used for the variable of time spent on Facebook. Based on the regression models, we computed and reported the marginal effects on Facebook use and time spent of adults age groups, including age groups 18-29, 30-49, 50-64, and 65 and over. We discuss these findings in the context of the implications for promoting positive health behaviors.

**Results:**

The demographics of the final sample (N=730) included adults age 18-65 years and above (mean 48.2 yrs, SD 18.3 yrs). The majority of the participants were female (372/730, 50.9%), white (591/730, 80.9%) and non-Hispanic (671/730, 91.9%). Bivariate analysis indicated that Facebook users and nonusers differed significantly by age group (χ^2^=76.71, *P*<.001) and sex (χ^2^=9.04, *P*=.003). Among subjects aged 50 and above, the predicted probability was 66% for spending the same amount of time, 10% with increased time, and 24% with decreased time.

**Conclusions:**

The key findings of this study were Facebook use among midlife and older adults was more likely to stay the same over time, compared to the other age groups. Interestingly, the young adult age group 18-29 years was more likely to decrease their time on Facebook over time. Specifically, younger females were most likely to decrease time spent on Facebook. In general, male participants were most likely to spend the same amount of time on Facebook. These findings have implications for future health intervention research, and ultimately, for translation to the clinic setting to improve health outcomes.

## Introduction

### Background

Social media has evolved rapidly and has been used as a means of communication, social interaction, and social support with minimal consideration for wider use in health care. It is recognized that social influences are an important factor in the adoption of health behaviors [1,2]. Despite the growing interest in practical research and the development and implementation of health behavior interventions in real-world settings, social media is often overlooked as an effective method to deliver the active components of a health behavior intervention. To accomplish this requires knowledge of the characteristics of social media use among the population of interest for integration in a health behavior intervention.

### Significance

The use of the social media is deeply embedded in everyday life for an increasing number of adults [3]. The widely recognized, accessed, and the culturally integrated Facebook platform has an extensive reach, with over one billion daily adult users and continues to grow [4].

The Facebook platform has been used effectively to recruit participants for health behavior research [5], to provide online health information and education [6,7] as well as the more familiar social networking functions. This platform has been used as an adjunct to multimodal interventions or in conjunction with investigator-initiated in-person visits and other methods of communication, such as mobile phones, text messages, and apps [8-11]. Facebook used as a means for patients to communicate with other patients is a trend referred to as peer-to-peer health care [12]. These functions of Facebook are widely recognized; however, peer support is only a small part of the potential of this platform to improve health outcomes.

Facebook offers the capability to create groups via the platform referred to as “Facebook Groups.” This group platform offers a convenient, reliable, private, members-only site that can be adapted to motivate and support health behavior change interventions and conduct research. This platform also allows integration into clinic processes, with health care professionals serving as moderators and contributors of the content to ensure its’ veracity. Efficacious health behavior interventions translated into real-life settings for effectiveness testing require the ability to appeal to and influence a broad and diverse population of participants. This necessitates an understanding of the characteristics of Facebook use of potential participants from the population of interest.

A distinct advantage of using the Facebook platform to facilitate a health behavior intervention is the high level of individual engagement. This is an important factor in promoting positive health behavior change. Engagement in an intervention is critical for participants to receive an adequate dose of the intervention [13], and thus, improve health outcomes. Attrition is very low on this platform, although some individual users occasionally take breaks from social media use [4]. Minimizing attrition may support long-term maintenance of health behaviors. The high levels of engagement and retention on Facebook over time may translate to the Facebook Groups platform making this an appealing method for delivering the active components of a health behavior intervention. Interventions facilitated by social media platforms hold promise to reduce the challenges for the growing population of midlife adults to prevent or delay the progression of chronic health conditions [14].

Few studies reported health behavior interventions facilitated by social media platforms to provide the active components of an intervention. A review found positive outcomes with 87.5% (7/8) of the studies involving physical activity interventions delivered using Facebook [15]. Other research found that Facebook user engagement and social support predicted a reduction in smoking [16]. Features of the Facebook Groups platform to promote cardiac rehabilitation included participant only access with a health care provider involved as group moderator to provide evidence-based information and assure “trustworthiness” of the group [17]. Greater Facebook capability may increase willingness to participate in the Facebook Groups platform. Trends show that adults across the age continuum are capable users of this technology [18]. Further, participants may be more likely to engage on the Facebook Groups platform upon the recommendation of their health care provider. Earlier studies reported that some older adults had concerns about the usefulness of combining health resources with social media [19]. Social media use has advanced since its’ inception and is now being used by younger adults as well as older adults to influence health. Therefore, we investigated the characteristics of adult Facebook users to inform our future research on the Facebook Groups platform.

### Study Objective

The objective of this study was to investigate the use of the social media platform, Facebook by adults (aged 18-65 years and above). Knowledge is limited to the characteristics of Facebook use by adults across the age continuum, necessitating this investigation. Characteristics of Facebook users are important to understand to guide the development of a future clinical trial using the social media platform, Facebook Groups to deliver the active components of a health behavior intervention.

## Methods

### Design

The Institutional Review Board at the University where this study was conducted approved this study as exempt from full board review due to the use of de-identified data. The design was a secondary analysis of survey data. The sample included adults across the age continuum (18-65 years and above). Participants were included in our analysis based on the following: (a) they responded “yes” to the initial question: “Do you use the internet?” (b) they were able to provide informed verbal consent by phone, and (c) they were able to speak and understand English or Spanish, as the survey was not conducted in any other languages.

### Data Collection

The Pew Research Center surveyed a representative sample of adults across all regions of the continental United States about their use of the social media platform, Facebook [20]. This national self-report survey was conducted between November 14, 2012 and December 9, 2012, on landlines and cell phones, and in English and Spanish by Princeton Survey Research Associates International. Multiple attempts were made to reach each phone number at a variety of times throughout the day. The survey consisted of 20 items rated on a Likert-type scale or a true and false format. We selected the sociodemographic and Facebook use the variables: user or nonuser, and time spent using Facebook stayed about the same, increased or decreased for this analysis.

### Data Analysis

We described demographics by adults’ use of Facebook and time spent on Facebook, using chi-squared tests for categorical variables. Fisher’s exact test was used for two variables due to small cell counts as noted. Multivariate binomial logistic regression was performed for the model of Facebook use (users and nonusers) on age group, controlling for other demographics, including sex, income, employment, race and ethnicity, and education. A multivariate, multinomial logistic regression model was used for our secondary outcome variable, regressing time spent on Facebook (stayed about the same, increased, and decreased) on age groups and other demographic variables. We also questioned whether the effects of age group on Facebook use and time spent would differ by sex. Therefore, after performing multivariate regression, we calculated marginal effects to show the interactions between age group and sex in the models. The marginal effects were interpreted as the predicted probability of using Facebook or time spent on Facebook for participants of a specific age group and sex, holding all other demographics constant to the means. We discuss these findings in the context of the implications for health behavior change. All data were analyzed using the STATA Version 14.0 (StataCorp, LP, College Station, TX, USA).

## Results

The demographics of the sample (N=730) are shown in Table 1 and included adults age 18-65 years and above with a mean of 50.3 (SD 19.7) years. The majority of the participants interviewed were white (591/730, 80.9%) and non-Hispanic or another ethnicity 91.9% (671/730). There were 50.9% (372/730) females and 49.0% (358/730) males responding to the survey. Some college or college graduation was reported by 69.0% (504/730) of the respondents. Half (365/730) of the sample reported full-time employment, with just over half (380/730, 52.0%) reporting an income between US $30,000 and US $100,000. The majority (462/730, 63.2%) surveyed were Facebook users, and 52.0% (380/730) reported their time spent on Facebook stayed the same over the past year.

The bivariate analysis (Table 2) indicated that Facebook users and nonusers differed significantly by age group (N=730, *χ*^2^_3_=76.7, *P*<.001), and sex (N=730, *χ*^2^_1_=9.0, *P*=.003). There were no significant differences in Facebook users and nonusers based on income, employment status, race and ethnicity, or education level.

The bivariate analysis (see Table 3) indicated that time spent on Facebook (ie, stayed about the same, increased, and decreased) differed significantly by age group (n=461, *χ*^2^_6_=31.8, *P*<.001). No differences were found in time spent on Facebook based on income, employment status, race, or education. Notably, ethnicity was significant (n=461, *χ*^2^_2_=5.3, *P*=.045) using Fisher’s exact test.

**Figure 1:**
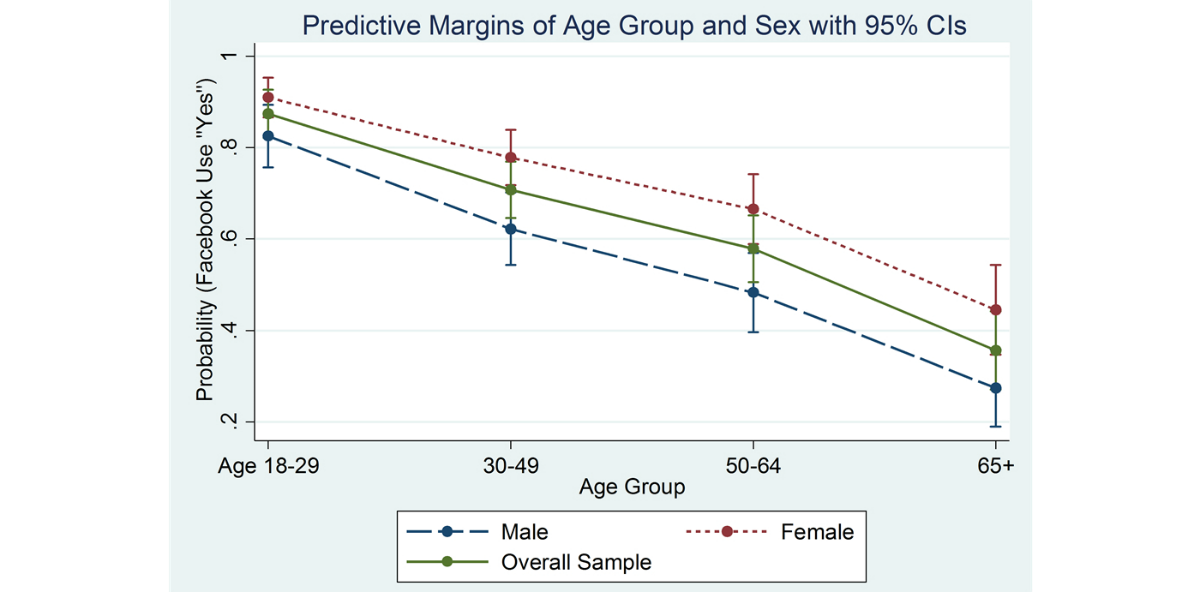
Marginal effects of age group and sex on Facebook use.

Based on our bivariate results, we examined whether the effects of age group differed by sex. Table 4 shows significant variations in the marginal effects of age group by sex on Facebook use. Younger and female subjects were more likely to use Facebook than older and male subjects. The probability of using Facebook was 87% among participants aged 18-29 years and differed by sex. The probability of using Facebook among participants aged 18-29 was 91% for female, whereas it was 83% for male. The probability of using Facebook among participants aged 65 and over was 45% for female, whereas it was 27% for male. Figure 1 shows the marginal effects of age group by sex on Facebook decreasing across age groups.

Table 5 shows marginal effects of age group and sex across the three subgroups of Facebook time (stayed the same, increased, and decreased). Due to small cell counts in age group 65+ and Asian group (Table 3) we regrouped the age variable into three groups (18-29, 30-49, and 50+) and the race variable into three groups (white, black or African American, and others) for analysis of time spent using Facebook.

Overall, subjects in the group that increased amount of time on Facebook showed the lowest probabilities than those in the other groups. Among subjects aged 50 and above, the predicted probability was 66% for spending the same amount of time, 10% with increased time, and 24% with decreased time. Older subjects were more likely to spend the same amount of time and less likely to decrease time on Facebook. Younger participants were more likely to decrease time on Facebook.

**Figure 2:**
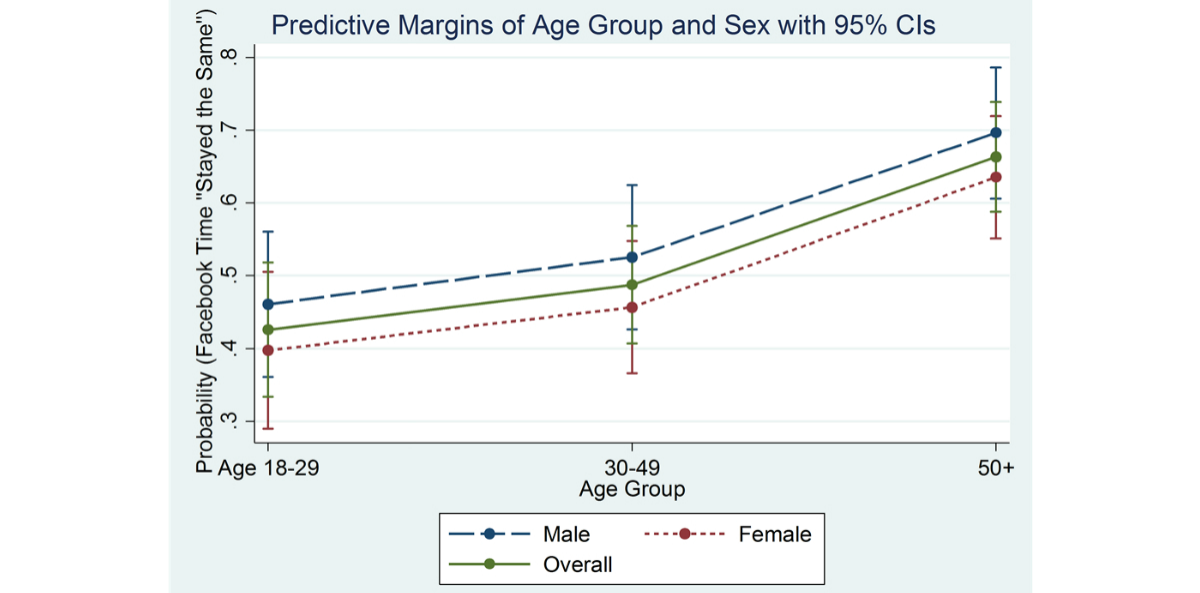
Marginal effects of age group and sex by Facebook time "Stayed the Same".

**Figure 3:**
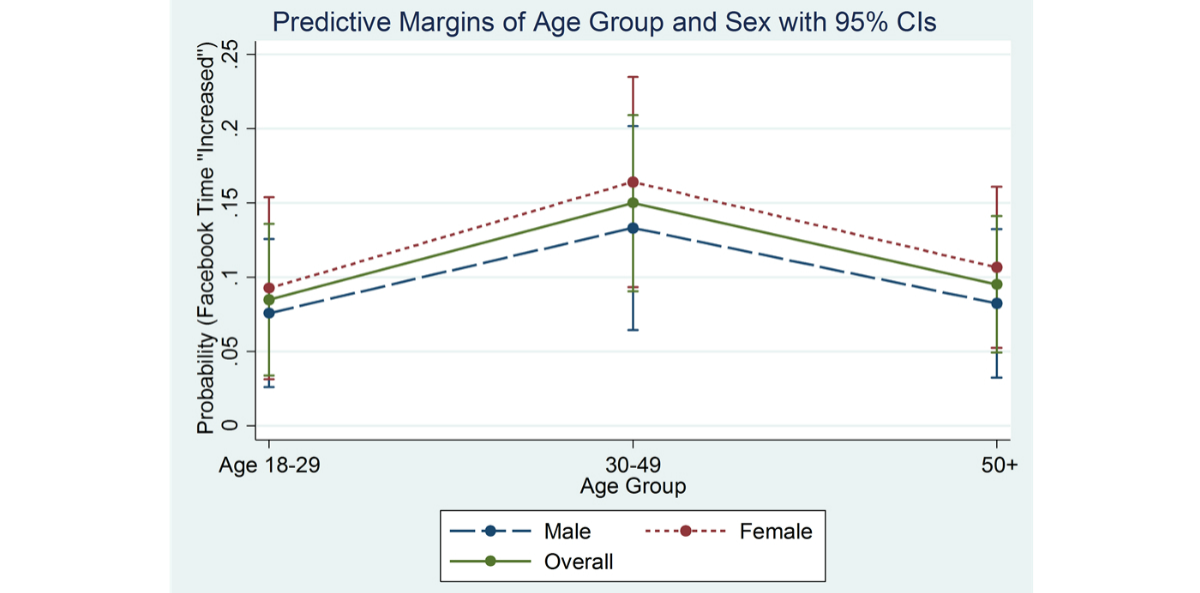
Marginal effects of age group and sex by Facebook time "Increased".

**Figure 4:**
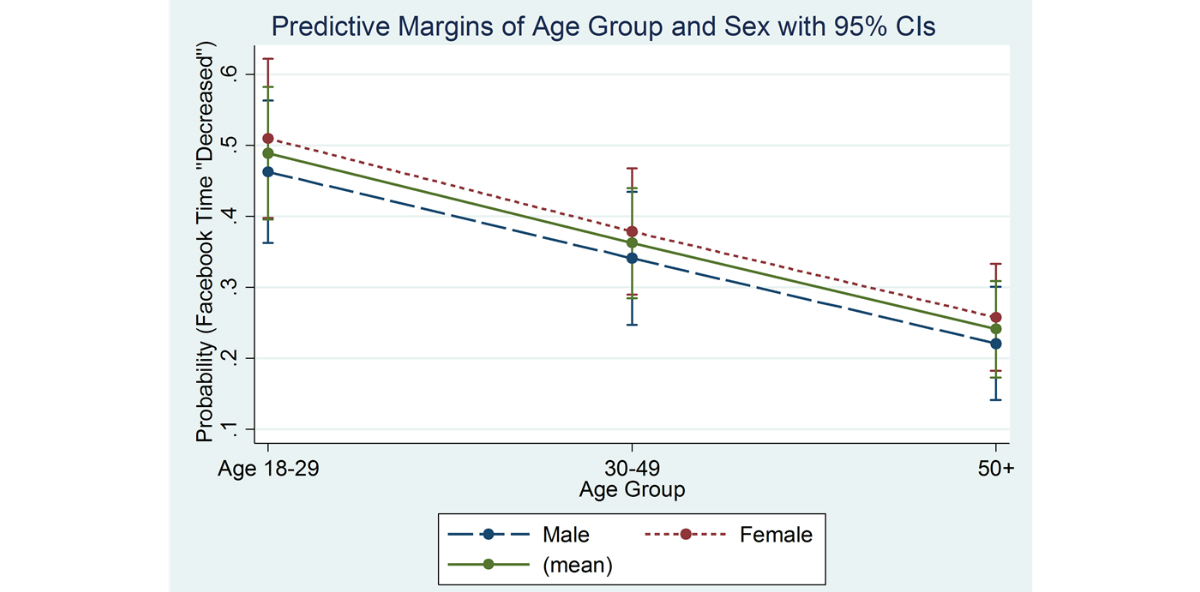
Marginal effects of age group and sex by Facebook time "Decreased".

Among older males, the predicted probabilities for time spent using Facebook were 70% stayed the same, 8% increased, and 22% decreased time on Facebook. The predicted probabilities for Facebook use among women in this age group were similar with 64% stayed the same, 11% increased, and 26% decreased time spent on Facebook. Figures 2-4 show the marginal effects of age group by sex on Facebook use for the variables: stayed about the same (Figure 2), increased (Figure 3), and decreased (Figure 4).

**Table 1:** Descriptive summary of demographic characteristics and outcome variables.

Variables		Value (N=730), n (%)
**Age group (years)**		
	18-29	152 (20.8)
	30-49	232 (31.8)
	50-64	191 (26.2)
	65+	155 (21.2)
**Sex**		
	Male	358 (49.0)
	Female	372 (51.0)
**Income (US$)**		
	<$30,000	219 (30.0)
	$30,000-$100,000	380 (52.1)
	>$100,000	131 (17.9)
**Employment**		
	Full-time	362 (49.6)
	Part-time	104 (14.2)
	Not employed	264 (36.2)
**Race**		
	Caucasian	589 (80.7)
	African-American	77 (10.5)
	Asian or Pacific Islander	19 (2.6)
	Other	45 (6.2)
**Ethnicity**		
	Hispanic	61 (8.4)
	Not Hispanic or other	669 (91.6)
**Education**		
	≤High school	223 (30.5)
	≥College	507 (69.5)
**Facebook users**		
	Users	462 (63.3)
	Nonusers	268 (36.7)
**Time spent on Facebook^a^**		
	Stayed the same	382 (52.3)
	Increased	96 (13.2)
	Decreased	252 (34.5)

^a^n=461. This subgroup was from Facebook users only and those responding to the item of time spent on Facebook.

**Table 2:** Bivariate statistics by Facebook users and nonusers.

Variable		Full sample (N=730), n (%)		Facebook users (N=462), n (%)		Facebook nonusers (n=268), n (%)		Chi-square (df)		*P* value	
**Age group (years)**								76.7 (3)		<.001	
	18-29		152 (20.8)		129 (27.9)		23 (8.6)				
	30-49		232 (31.8)		163 (35.3)		69 (25.7)				
	50-64		191 (26.2)		109 (23.6)		82 (30.6)				
	65+		155 (21.2)		61 (13.2)		94 (35.1)				
**Sex**								9.0 (1)		.003	
	Male		358 (49.0)		207 (44.8)		151 (56.3)				
	Female		372 (51.0)		255 (55.2)		117 (43.7)				
**Income (US $)**								0.6 (2)		.748	
	< $30,000		219 (30.0)		140 (30.3)		79 (29.5)				
	$30,000-$100,000		380 (52.1)		236 (51.1)		144 (53.7)				
	>$100,000		131 (17.9)		86 (18.7)		45 (16.9)				
**Employment**								5.6 (2)		.062	
	Full-time		362 (49.6)		237 (51.3)		125 (46.6)				
	Part-time		104 (14.2)		73 (15.7)		32 (12.0)				
	Not employed		264 (36.2)		153 (33.1)		111 (41.4)				
**Race**								6.9 (3)		.076	
	White		589 (80.7)		361 (78.1)		228 (85.0)				
	Black or African-American		77 (10.5)		57 (12.3)		20 (7.5)				
	Asian or Pacific Islander		19 (2.6)		15 (3.3)		4 (1.5)				
	Other		45 (6.2)		30 (6.4)		16 (6.0)				
**Ethnicity**								3.2 (1)		.076	
	Hispanic		61 (8.4)		45 (9.7)		16 (6.0)				
	Not Hispanic (other)		669 (91.6)		417 (90.3)		252 (94.0)				
**Education**								1.0 (1)		.307	
	≤High school		223 (30.5)		135 (29.2)		88 (32.8)				
	≥College		507 (69.5)		327 (70.8)		180 (67.2)				

**Table 3:** Bivariate statistics by time spent on Facebook (n=461).

Variable		Stayed about the same (n=241), n (%)	Increased (n=61), n (%)	Decreased (n=159), n (%)	Chi-square (df)	*P* value
**Age group (years)**					31.8 (6)	<.001^a^
	18-29	55 (22.8)	12 (19.7)	61 (38.4)		
	30-49	75 (31.1)	29 (47.5)	59 (37.1)		
	50-64	65 (27.0)	17 (27.9)	27 (17.0)		
	65+	46 (19.1)	3 (4.9)	12 (7.5)		
**Sex**					1.5 (2)	.466
	Male	112 (46.5)	23 (37.8)	72 (45.3)		
	Female	129 (53.5)	38 (62.3)	87 (54.7)		
**Income**					7.4 (4)	.118
	<$30,000	67 (27.8)	16 (26.2)	56 (35.2)		
	$30,000-$100,000	134 (55.6)	28 (45.9)	74 (46.5)		
	>$100,000	40 (16.7)	17 (27.9)	29 (18.3)		
**Employment**					5.3 (4)	.255
	Full-time	123 (51.0)	26 (42.6)	88 (55.4)		
	Part-time	32 (13.3)	13 (21.3)	26 (16.4)		
	Not employed	86 (35.8)	22 (36.1)	45 (28.3)		
**Race**					6.4 (6)	.363^a^
	White	196 (81.3)	46 (75.4)	118 (74.2)		
	Black or African-American	24 (10.0)	7 (11.4)	26 (16.4)		
	Asian or Pacific Islander	6 (2.5)	4 (6.7)	5 (3.1)		
	Other	15 (6.2)	4 (6.7)	10 (6.4)		
**Ethnicity**					5.3 (2)	.045^a^
	Hispanic	27 (11.2)	1 (1.6)	17 (10.7)		
	Not Hispanic (Other)	214 (88.9)	60 (98.4)	142 (89.3)		
**Education**					3.2 (2)	.204
	≤High school	75 (31.1)	12 (19.7)	48 (30.2)		
	≥College	166 (68.9)	49 (80.3)	111 (69.8)		

^a^*P* values from Fisher’s exact test due to small cell counts.

**Table 4:** Marginal effects of age group and sex on the probability of using Facebook.

Variable			Facebook user^a^, respondent probability (95% CI)	*P* value
**Age group (years)**				
	18-29		0.87 (0.82-0.92)	<.001
	30-49		0.71 (0.65-0.77)	<.001
	50-64		0.58 (0.51-0.65)	<.001
	65+		0.36 (0.27-0.44)	<.001
**Sex**				
	Male		0.56 (0.51-0.62)	<.001
	Female		0.73 (0.69-0.78)	<.001
**Age group by sex (years)**				
	**Male**			
		18-29	0.83 (0.76-0.89)	<.001
		30-49	0.62 (0.54-0.70)	<.001
		50-64	0.48 (0.40-0.57)	<.001
		65+	0.27 (0.19-0.36)	<.001
	**Female**			
		18-29	0.91 (0.87-0.95)	<.001
		30-49	0.78 (0.72-0.84)	<.001
		50-64	0.67 (0.59-0.74)	<.001
		65+	0.45 (0.35-0.54)	<.001

^a^Marginal effects from binary logistic regression, adjusted for income, employment status, ethnicity, race, and education.

**Table 5:** Marginal effects of age group and sex on the probability of time on Facebook.

Variable			Stayed about the same^a^, respondent probability (95% CI)	*P* value	Increased^a^, respondent probability (95% CI)	*P* value	Decreased^a^, respondent probability (95% CI)	*P* value
**Age group (years)**								
	18-29		0.43 (0.33-0.52)	<.001	0.08 (0.03-0.14)	.001	0.49 (0.40-0.58)	<.001
	30-49		0.49 (0.41-0.57)	<.001	0.15 (0.09-0.21)	<.001	0.36 (0.28-0.44)	<.001
	50+		0.66 (0.59-0.74)	<.001	0.10 (0.05-0.14)	<.001	0.24 (0.17-0.31)	<.001
**Sex**								
	Male		0.58 (0.50-0.65)	<.001	0.10 (0.05-0.14)	<.001	0.32 (0.26-0.39)	<.001
	Female		0.51 (0.44-0.58)	<.001	0.12 (0.08-0.17)	<.001	0.37 (0.30-0.43)	<.001
**Age group by sex (years)**								
	**Male**							
		18-29	0.46 (0.36-0.56)	<.001	0.08 (0.03-0.13)	.003	0.46 (0.36-0.56)	<.001
		30-49	0.53 (0.43-0.63)	<.001	0.13 (0.06-0.20)	<.001	0.34 (0.25-0.43)	<.001
		Age 50+	0.70 (0.61-0.79)	<.001	0.08 (0.03-0.13)	.001	0.22 (0.14-0.30)	<.001
	**Female**							
		18-29	0.40 (0.29-0.51)	<.001	0.09 (0.03-0.15)	.003	0.51 (0.40-0.62)	<.001
		30-49	0.46 (0.37-0.55)	<.001	0.16 (0.09-0.24)	<.001	0.38 (0.29-0.47)	<.001
		50+	0.64 (0.55-0.72)	<.001	0.11 (0.05-0.16)	.001	0.26 (0.18-0.33)	<.001

^a^Marginal effects from multinomial logistic regression, adjusted for income, employment status, ethnicity, race, and education; 95% confidence intervals in parentheses.

## Discussion

### Principal Findings

The key findings of this study were Facebook use among midlife adults was more likely to stay the same over time, compared to the other age groups. Interestingly, in the young adult age group, 18-29 years was more likely to decrease their time on Facebook over time. Based on the available data, we are not able to explain this finding but it may be due to the use of other competing social media platforms (eg, Twitter, Instagram, Snapchat, etc) and lifestyle factors unique to younger adults. Younger females were most likely to decrease time spent on Facebook. In general, male participants were most likely to spend the same amount of time on Facebook. The probability of increasing time on Facebook was low for both males and females.

Earlier reports indicated that midlife and older adults were unlikely to use social media to seek or discuss health information as it was perceived as an intrusion of privacy [21]. This age group was reportedly reluctant to learn about using the latest technology [3]. Our study findings contradict these perceptions and show that older adults had the highest probability of maintaining Facebook use over time. Our results demonstrated that older adults’ use of Facebook stayed about the same over time, compared to decreased time in the younger age adult groups.

The reluctance of adults of any age to participate in social media may be related to a lack of awareness or familiarity with the technology. Facebook is the most widely used social media platform, and the user base has grown more representative of the broader population [18]. While young adults were among the earliest social media adopters and had high rates of social media use, use among midlife and older adults has increased considerably in recent years [18]. An update of the Pew Research Center survey data reported in early 2018 indicated that ownership of mobile phones used to access social media platforms has continued to grow to nearly three-quarters of adults in the United States [22]. In this update, adults age 18 to 49 years were found to be close to saturation adoption. This is nearly double the percentage measured in the Pew survey in 2012. Notably, the fastest growing demographic regarding Facebook use was with the midlife and older adult population.

The Facebook Groups platform with high levels of engagement, low levels of attrition over time, and wide reach holds promise to motivate and support health behavior change for the growing population of midlife adults. Providing technical assistance for midlife and older adults with mobile device use and navigating the Facebook Groups platform must be considered to improve use for this age group when developing health behavior interventions.

Considerations for generalizing these findings to other populations include the representative sample of adults within the continental United States and the global reach of the Facebook platform. The wide geographical sampling distribution strengthens the Pew Research Center data for generalization to US adults, but may not be representative of the global population. We also acknowledge that secondary analyses of existing data are limited to the variables in the dataset, and this can be considered a limitation. We acknowledge that the variables used in this analysis may not be sufficient to account for other unmeasured factors related to our outcome variables. However, the sociodemographic variables examined, Facebook use and nonuse variables, and Facebook use over time support our study objective.

### Conclusion

Overall, the findings of this study point to considerations for researchers to use the Facebook Groups platform to facilitate health behavior interventions. In addition to social communication and interaction, this platform is capable of providing health information, motivation and support for positive health behavior change. This platform can be integrated with clinic processes, involving health care professionals as moderators and contributors to ensure the veracity of the content. Providing a health behavior intervention on a social media platform is a unique approach to improve health outcomes, specifically, with the large and growing midlife adult population with escalating risk for chronic conditions. Our data indicate more consistent Facebook use among midlife adults than younger adults. Prospective research is needed to determine if the levels of engagement and retention of Facebook use for general social networking purposes translates to health behavior interventions facilitated by the Facebook Groups platform.
